# Emerging Zika virus infection and bioethical considerations: Comment to: Bioethical considerations at times of Zika virus. Einstein (Sao Paulo). 2016;14(2):13-8

**DOI:** 10.1590/S1679-45082016CE3822

**Published:** 2016

**Authors:** Viroj Wiwanitkit

**Affiliations:** Surin Rajabhat University, Surin, Thailand

Dear Editor,

The recent article on “Bioethical considerations at times of Zika virus”^(1)^ is very interesting. As a new emerging disease, undoubtedly there is no clear information on the disease and use of diagnostic tests. Therapeutic options or preventive measures can be considered risks, leading to ethical issues. In fact, it is hard to manage the situation of any new emerging infection. Sometimes, the use of unproved treatment, such as drugs and vaccines, is required to meet the demands of a rapidly progressing outbreak.^(2)^ The recent case of Ebola outbreak is a good example of a lesson learnt.^(3)^ Nevertheless, since many patients infected with Zika virus are mild and asymptomatic cases,^(4)^ the use of not proven novel therapy has to be carefully considered. The great challenge might be the pregnant subjects, and managing the cases is usually an ethical challenge.^(5)^ The next issue to be discussed might be induction of abortion. This can be an ethical dilemma and a big concern in terms of religions. There are also other ethical issues regarding the new infection that are not often mentioned. Transparency when disclosing information to the general public is a big concern. In some countries, the data might be blinded in order to control panic of the local people. In addition, medical staff and medical scientists should share data.^(6)^ Sometimes the medical business patent might override the humanistic concern in management of worldwide health crisis. The very high cost of a newly developed drug is expected and how to manage this future problem should be thought. Finally, the risk run by medical teams should not be forgotten. Promoting safety at workplace, including good vector control in the medical center must be the basic requirement set by policy makers and administrators.
